# The Study of Languages

**Published:** 1910-09-03

**Authors:** 


### THE STUDY OF LANGUAGES.

### ITS IMPORTANCE TO THE MEDICAL STUDENT.

When it was customary for medical men to write their prescriptions, and not only their prescriptions but their case books as well, in Latin, every medical practitioner could at least boast that he had a smattering of another language besides his vernacular. Latin in those days was the international medium of scientific communication. It held a position which no other language is likely to attain in the near future, and its study afforded an excellent means of inculcating precision of expression and, to some extent, accuracy of thought. Who, reading the letters of Boerhaave preserved in the Sloane MSS. at the British Museum, can doubt that the man who was able to write so clearly in an acquired tongue merited fully the title of the most lucid lecturer of his day? Boerhaave's case is no exception. His contemporaries were equally at home in at least two languages, and it is reasonable to argue that the insignificance of modern medical literature, from a purely literary point of view and when contrasted with the classical writings in medicine, is partly due to the fact that modern medical authors have not learned to think in more than one.

### The Practical Value of Language Study.

It is useless and needless here to dilate upon the general advantages of the study of foreign languages. The student who has not already sufficiently realised these advantages, if only in theory, is not likely to be touched by their general analysis. But it is fully worth while to point out the particular advantages which the student of medicine who is acquainted with some other language beside his own possesses over his less lucky or less energetic fellows to whom everything beyond English is foreign and incomprehensible. It is as well that the student should know these at the beginning of his career. There are many studies that will claim his attention as he progresses in his work. The attractions of purely scientific study, whether clinical or in the laboratories, will appeal to him strongly after his first two years, and a diligent application to his duties in the wards will ordinarily leave him little leisure and, unless he is exceptionally energetic or ambitious, little inclination to take up the study of a foreign language, once he has passed through his second professional examination. Once qualified and in the ruck of general practice, his opportunities for such a study may, of course, be as he likes to make them; but then again it is likely that the drudgery and weariness of making a competence—we will not say amassing a fortune—will distract his attention from the sober, sedentary pursuit of philology. Therefore the best chance for success lies in his earlier years, when the press of work is not so heavy and the opportunities for excursions into other fields beyond his own immediate scientific acre are greater than later on. It is possible that many will find the grind at the preliminary subjects abominable enough, and argue that there is little time comfortably to get through with biology, chemistry, anatomy, and physiology. A hard day in the dissecting-room and a couple of hours over Gray and Halliburton do not leave much time for further distraction. But the best relaxation, mental as well as physical, is found not in absolute rest, but in variation. A tussle with Cervantes in the original after a bout with the ganglia of the fifth, comes as a tonic and a blessing almost, and to those who have never tried the treatment we can only commend it.

### Classical Languages.

From a purely practical point of view the study of a foreign language is to be advised to the medical student, not only because it will enlarge his mental purview, strengthen his memory, broaden his vocabulary and his ideas alike, and add immeasurably to his knowledge of his own language, but more especially because nowadays it is imperative for the medical man who wishes to keep abreast of the developments of his science to know either French or German. No man can conscientiously undertake post-graduate study abroad unless he has an acquaintance with the language of the country where he proposes to work. With regard to classical languages every student has a smattering of Latin. So much at least the preliminary arts require of him, but in nine cases out of ten it goes no further. A doctor who is a classical scholar is in these days the exception, even when one takes the term in its limited sense as implying one who has a good knowledge of Latin and Greek. Syriac, Hebrew, and Arabic, like Logic—once included in the curriculum of the specialist in medicine—are now absolutely out of court and have been for the last half-century, although enthusiasts like the learned Dr. Adams attempted to keep the flame of philological classicism alive in the faculty. The want of an adequate knowledge of Latin is seen daily in the ludicrous mistakes made in prescription-writing by those who still attempt to follow the ancient rules of the art, and also in the equally ludicrous, if more excusable, ignorance of the meaning of familiar professional terms. The study of the classics should be encouraged if only for the reason that they will enable students to realise the absurdity of some of our medical nomenclature and the execrable new coinages that have lately been introduced among us.

### Foreign Languages.

Entirely different is the case with foreign contemporary languages. We do not plead for the study of philology: only for such interest in a modern language as will enable a student to acquire a working knowledge of it. He will find such knowledge of inestimable value to him in after-life. It will put him into closest touch with his colleagues who write and speak in another language, and will enable him to be thoroughly independent when he decides to visit foreign hospitals and study outlandish methods. It is true nowadays a man can wander far afield armed merely with English and a small amount of self-confidence, but he runs the risk of misunderstanding and being misunderstood, and loses half of the profit he may gain from a *Studienreise* if he possesses nothing else but these two stand-bys.

### The Best Languages to Study.

The most important modern languages from a practical point of view for the medical student to master are undoubtedly French and German; a knowledge of the latter more especially is fast becoming a necessity to the specialist. Italian is of less importance from a professional point of view, though much good work is being done in Italy, the records of which are to be found in the numerous Italian professional journals. It possesses, however, the advantage of being a comparatively easy language to learn, and of opening to the student a wealth of literature which will unfailingly attract by its interest and originality. Russian, which already possesses a vast medical and scientific literature, is, on the other hand, an intensely difficult and troublesome language to acquire, and it is questionable whether the bother of conquering its prodigious alphabet and struggling with its intricate, irregular verbs is compensated ultimately by the use which the student can make of his knowledge. Spanish and Portuguese are of value to those who are specially interested in tropical medicine, and, like Italian, are fairly easy to master, while the general literature of both is extremely interesting. The other European languages offer little inducement to the medical student unless he has some special object in view. Those who desire to join the Indian Medical Service will of course find one of the Oriental languages of great value. Persian, modern Arabic, Hindustani, and Malay are all useful, and once the difficulty of the strange and novel caligraphy is overcome do not offer any serious obstacles to the persevering student. But the two chief languages—one of which medical students, no matter what they desire to do when qualified, will do well to tackle—are French and German.

### What is Wanted.

To become a proficient scholar in either of these, as in any language, demands an amount of care and attention, patience and time, which few professional men can afford to give; but few of us can hope to become proficient in more than one tongue—the majority hardly ever get even so far! For all practical purposes a working knowledge of the language is sufficient; it enables one to read, and if necessary to correspond, in the language, to talk, and to understand it without difficulty, if not with ease. The importance of this qualification is great, but is often lost sight of. Many a man can read and enjoy a French author without being able to explain why the latter uses certain words and without perhaps being able to give a literal translation of the passages he has read. That is the amount of knowledge necessary to acquire and with which most students can afford to be content.

### How to Study.

How, then, is it possible to acquire this working knowledge? Obviously the best and easiest course is to reside in a foreign country where the tongue is spoken, and to acquire it by intercourse with the natives. That, however, is impossible in the circumstances, and the student must make shift with the substitutes that are offered him at home. He can attend classes at the various schools, special and otherwise. Of these there are many in London, and it would be invidious to single out any one of them, although it must be said that not all of them are equally good, while most are expensive. A card to the principal of any of these institutions, stating the exact requirements of the student, will always bring a courteous reply supplying full information, and usually classes are arranged to suit the time of individual students. Private tuition is always available, though it costs a trifle more than the ordinary class fees. The student who wishes to be independent can advertise for a teacher or for conversation lessons, and may often in that way get very good instruction at a comparatively cheap rate, the fees varying from 2s. upwards per hour. In such cases the school or teacher will supply all the information necessary, but he cannot guarantee a success unless the student is diligent and works at the subject. And in the study of languages self-help is the great thing. An energetic student can educate himself very thoroughly. For pronunciation and idiom he requires, of course, oral lessons, but there are many ways of obtaining them, even in London. A dinner at a small Soho restaurant or a purchase at a Wardour Street dealer's shop should prove a valuable lesson to the student who is not shy and who is really desirous of getting on. A good grammar and an equally good dictionary are necessaries, and we would strongly urge upon the student the desirability of attempting to read the language he decides to learn from the very start. The easy translation extracts given in most manuals are comparatively valueless. A daily attempt with a newspaper or a simple story is of much greater use, and the student should subscribe from the first to one or other periodical. For students who prefer to work alone, the excellent method of the Hugo school is to be commended. The subscription to the paper issued by the school is very low, and its course is one of the best that we know of. There are many simple tales and stories which the learner can commence with, and some of these are comparatively little known—such as, for instance, the criminal *romans* in German and the "historic" sensational novels sold in the Paris kiosks and to be obtained in the Soho book-shops! These may not be literature—we do not say they are—but they are mildly interesting and their dialogue is always bright and idiomatical. The well-known classics, a list of which is easily made out, furnish many other examples, but the student should get someone to advise him as to what classics to select. A start with "Nathan der Weise" or with the "Chants de Crépuscule" is not likely to be encouraging for the beginner in German or French, any more than a preliminary canter with the Paraphrase to the Psalms would be for a foreign student who commenced his study of English!

### Scientific Words.

The medical student will find it to his advantage to get a good scientific paper in the language he is bent upon learning and to devote a few hours of his time per week to it. At first no doubt it will frighten him badly—scientific German is awe-inspiring enough—but gradually as he gets his footing he will overcome his awe, and the knowledge gained of phraseology and professional terms will be invaluable. From time to time, too, the student should test his knowledge by talking or listening to others talking in the language he studies. A visit to the Continent in vacation time will greatly aid him and will be an invaluable adjunct to his studies.

The cost of such study, in time and money, it is difficult to estimate, since it must necessarily largely depend on the individual. The outlay in books in the case of the self-helper is comparatively slight—a few guineas ought to cover it handsomely. In the case of a class student, or one who employs a private coach, the fees will of course amount to a good deal more; but here again the expenditure ought not to be excessive. As to time, a few hours every week devoted to the study, with a few minutes' daily repetition or reading, should produce good results in three months' time in any student who has an interest in the subject. After that progress is a matter of application and practice.

